# Chronic Rheumatologic Disease in Chikungunya Virus Fever: Results from a Cohort Study Conducted in Piedecuesta, Colombia

**DOI:** 10.3390/tropicalmed9100247

**Published:** 2024-10-19

**Authors:** Anyela Lozano-Parra, Víctor Herrera, Carlos Calderón, Reynaldo Badillo, Rosa Margarita Gélvez Ramírez, María Isabel Estupiñán Cárdenas, José Fernando Lozano Jiménez, Luis Ángel Villar, Elsa Marina Rojas Garrido

**Affiliations:** 1 Grupo Epidemiología Clínica, Escuela de Medicina, Universidad Industrial de Santander UIS, Calle 9 Carrera 27, Bucaramanga 680002, Colombia; anyela.lozano@correo.uis.edu.co (A.L.-P.); vicmaher@uis.edu.co (V.H.); 2 Centro de Atención y Diagnóstico de Enfermedades Infecciosas (CDI), Fundación INFOVIDA, Cra. 37 No. 51-126, Bucaramanga 680003, Colombia; cacalderonco@unal.edu.co (C.C.); coordinacioninvestigacion@cdi.net.co (R.M.G.R.); maria.estupinan@correo.uis.edu.co (M.I.E.C.); jose.fernando.lozano@correounivalle.edu.co (J.F.L.J.); direccioninvestigacion@cdi.net.co (L.Á.V.); 3 Departamento Medicina Interna, Universidad de Santander-UDES, Calle 35 # 10-43, Bucaramanga 680006, Colombia; dir.medicinainterna@udes.edu.co

**Keywords:** Chikungunya, chronic rheumatism, rheumatic disease, fatigue, quality of life

## Abstract

This study aimed to determine the incidence of post-chikungunya chronic rheumatism (pCHIK-CR) and its impact on quality of life (QoL) and chronic fatigue in adults seven years after the 2014–2015 CHIKV outbreak in Piedecuesta, Colombia. We evaluated 78 adults (median age: 30 years, IQR: 21.0; women 60.3%) with confirmed CHIKV infection. In 2022, participants underwent a GALS examination and completed surveys on disability, stiffness, health status, and fatigue. A rheumatologist evaluated patients who reported arthralgia, morning stiffness, and abnormal GALS examination. Chronic fatigue was defined as fatigue persisting for over six months. Seven years after infection, 14.1% of participants were classified as pCHIK-CR cases, 41.0% as having non-inflammatory pain, likely degenerative (NIP-LD), and 44.9% without rheumatic disease (Wo-RM). Patients with pCHIK-CR and NIP-LD exhibited significantly worse QoL compared to Wo-RM cases. Chronic fatigue prevalence increased from 8.6% in Wo-RM patients to 25.0% in NIP-LD and 54.6% in pCHIK-CR cases. This study implemented a comprehensive clinical assessment to objectively estimate and characterize the incidence of chronic rheumatological disease attributed to CHIKV infection. One in seven cases with CHIKV infection develops pCHIK-CR, which impacts both QoL and chronic fatigue. This study contributes to understanding the burden of these arboviruses in the medium term.

## 1. Introduction

Chikungunya fever is a mosquito-borne disease caused by the chikungunya virus (CHIKV), an RNA virus member of the *Alphavirus* genus, the *Togaviridae* family [1]. Its genome is composed of two regions: the structural region encodes six proteins (capsid protein, envelope (E) E3-E2–6K-E1, and transferase protein), and the non-structural region encodes 4 proteins (nsP1, nsP2, nsP3, and nsP4) [2]. CHIKV are grouped into three genotypes: Asian, West African, and East-Central-South-African (ECSA) [3].

The emergence of CHIKV in the Americas is recent, causing about 3.7 million cases of infection between December 2013 and June 2023 [4]. In 2015, Colombia reported the highest number of infections in the Americas, with 275,907 cases [5]. However, a study conducted in 2017 in five Colombian cities found that CHIKV seroprevalence ranged from 9.0% to 73.0%, highlighting the heterogeneity in virus attack rates and susceptibility in specific populations to its reemergence [6,7].

Chikungunya fever is characterized by joint pain, which can sometimes be long-lasting and incapacitating. In addition to fever and joint pain, symptoms such as myalgia, headache, nausea, fatigue, and rash may appear during the acute stage (5–7 days) of the disease [8,9]. While most patients recover, some experience musculoskeletal pain, disabling joint pain, and fatigue for more than three months and even years [10]. In atypical cases, CHIKV could cause neurological manifestations such as encephalitis, Guillain-Barré syndrome, optic neuritis, and myelitis [11]. Different studies have found that post-CHIKV rheumatologic manifestations occur in 17.0% to 53.7% of cases, with variations reflecting differences in follow-up periods and the definitions for these complications [12,13,14]. Factors such as age over 45 years [15], female sex [16], history of joint pain [16], and high levels of IgG anti-CHIKV antibodies [15,16] predispose patients to the chronic form of the disease. CHIKV is not the only arthritogenic alphavirus; other related viruses, like Mayaro, Sindbis, Ross River, and O’nyong’nyong, have been associated with severe arthralgia and encephalitis [17,18]. Like other alphavirus, CHIKV has tropism for human synovial fibroblasts, endothelial cells, monocytes, and monocyte-derived macrophages [19].

During the acute phase of CHIK infection, high viral loads have been reported in human blood [20,21,22], eliciting the type I interferon (IFNα and IFNβ) response [20,23,24,25,26,27,28,29]. This leads to the production of pro-inflammatory cytokines and chemokines, such as IFN-α, IFN-γ, Il-2, IL-2R, IL-6, IL-7, IL-8, IL-12, IL-15, IL-17, IL-18, MCP-1, MIG, MIP-1α, MIP-1β, and IP-10 [20,22,29,30,31,32,33]. An in vivo study showed persistent viral antigens of CHIKV in macaques 90 days post-infection [34], potentially explaining the persistent joint symptoms. Furthermore, CHIKV’s nsP2 protein may inhibit the antiviral state IFN-dependent by blocking the JAK/STAT pathway, impairing the virus elimination [2] and contributing to rheumatologic manifestations [35,36,37]. MCP-1, MIG, IP-10, and IL-8 play key roles in recruiting immune cells such as macrophages, monocytes, NK cells, and T cells to affected joints, contributing to joint pain inflammation [18,31,38]. Moreover, MCP-1, IL-6, and IL-8 can promote the differentiation of monocytes into osteoclasts, stimulating bone resorption and worsening joint damage in CHIKV infection [18,39]. Post-CHIKV chronic rheumatism (pCHIK-CR) was first described in 1979 [40]. However, even after 10 years of CHIKV emergence in America and the efforts to characterize symptoms during the chronic phase of the disease, there is still no standardized and reproducible definition of pCHIK-CR. A proposed definition of pCHIK-CR is the persistence of joint and extra-articular symptoms for more than three months after the onset of CHIKV disease or the development of specific immune-mediated inflammatory pathology during follow-up [41,42].

The persistence of joint symptoms could impact the quality of life (QoL) [43,44,45,46]. This impact appears to be long-lasting and affects both mental and physical aspects of health, particularly concerning rheumatological manifestations [47]. Additionally, patients who recover from CHIKV tend to have a better quality of life compared to those with persistent symptoms [46]. Chronic fatigue is increasingly recognized as a symptom accompanying post-chikungunya rheumatic findings [48,49,50]. It has even been suggested that a significant proportion of musculoskeletal pain may be due to chronic fatigue rather than the primary rheumatic disease [48].

Considering the above, this study aimed to estimate the incidence of pCHIK-CR in adult patients seven years after the 2014–2015 CHIKV outbreak in Piedecuesta, Colombia. Additionally, we sought to determine the impact on quality of life and the presence of chronic fatigue associated with pCHIK-CR. Finally, we evaluated the effectiveness of the Health Assessment Questionnaire disability index (HAQ-DI) and the handgrip strength test for identifying pCHIK-CR in the chronic phase.

## 2. Materials and Methods

We used clinical and epidemiological data from two prospective cohort studies to identify cases of CHIKV infection, assessed the incidence of post-chikungunya chronic rheumatism (pCHIK-CR), and evaluated its impact on the quality of life and its association with chronic fatigue. These cohorts were initially designed to determine the etiology of acute febrile syndrome. This section describes both source studies, the procedure for data harmonization, the eligibility criteria, the recontacting protocol, participants’ follow-up, outcome definitions, and the analytical approach.

### 2.1. Source Studies

We identified acute CHIKV infection cases from two prospective cohorts conducted during the 2014–2015 outbreak in Piedecuesta, Colombia, as described below:

Passive surveillance cohort: This study was assembled in 2014 and included 839 patients aged 1–55 years who sought health care due to an unspecified acute febrile syndrome of less than or equal to a week of duration. The syndrome’s etiology was determined upon inclusion, and participants underwent a follow-up evaluation 7–14 days afterward [51].

Active surveillance cohort: This study was assembled in 2015 and included 2000 healthy children aged 2–15 years and 400 healthy adults who were identified through a convenience sampling from the community [7]. Participants underwent biweekly telephone follow-ups over 3.5 years to identify cases of acute febrile illness. They were evaluated within a week of the disease’s onset to determine etiology and followed 7–14 days later.

In both studies, the diagnosis of CHIKV infection was determined by a positive result in the NovaLisa^®^ IgG or IgM ELISA tests or the reverse transcription polymerase chain reaction (RT-qPCR) test [52]. We adopted this diagnostic approach because the virus was newly introduced to the population during the study period. We identified a total of 219 CHIKV infection cases: 188 from the passive surveillance cohort and 31 from the active surveillance cohort.

### 2.2. Data Harmonization

To harmonize the data from both cohorts, we initially reviewed their data dictionaries to assess the availability of relevant variables and their definitions. This process aimed to evaluate semantic reconciliation between the dictionaries and to establish harmonization rules. Finally, we syntactically transformed demographic and clinical variables to ensure they were suitable for data analysis.

### 2.3. Eligibility and Re-Contact

In this analysis, we included adult participants over 18 years old at follow-up visits who experienced an acute febrile illness and had a confirmed diagnosis of CHIKV infection. We excluded individuals with a history of any general physical or mental illness, those with any rheumatic disease diagnosed before the CHIKV infection, as well as those who did not consent to share their data or samples for future studies as per the informed consent obtained by the source cohorts. Eligible participants underwent a comprehensive re-contact protocol that included at least three phone calls (two on weekdays and one on weekends) made to every available telephone number in the databases, text messages through a short message service (SMS), a search on social networks (Facebook and Instagram), and at least an attempt to establish direct contact at their address of residency. After re-contacting and updating residency information, we excluded participants who resided outside the Metropolitan Area of Bucaramanga and those not interested in further evaluation.

### 2.4. Follow-Up

We invited eligible participants who were successfully re-contacted to attend a follow-up visit between September and December 2022. During this visit, participants provided informed consent and underwent a comprehensive physical examination focused on musculoskeletal health using the GALS methodology (Gait, Arm, Legs, Spine) [53,54], conducted by a physician who had been trained by a rheumatologist. Participants also performed a handgrip strength test following the guidelines of Roberts et al. [55] and answered the following self-reported questionnaires to assess their health status: the Health Assessment Questionnaire Disability Index (HAQ-DI) [56], the Musculoskeletal Stiffness Questionnaire (MSQ) [57], the SF-36v2 Health and Wellness Questionnaire [58], and the Fatigue Severity Scale (FSS) [59].

A certified rheumatologist conducted a second, independent evaluation on those participants who had any of the following findings in the first visit (median time between visits = 10 days): a report of arthralgia in one or more joints or morning stiffness lasting more than 30 min, an abnormal GALS examination, signs of joint swelling, enthesopathy or spondyloarthropathy non-trauma-related. The rheumatologist also evaluated a random sample of the participants with a normal first examination to estimate the rate of false negative results. Finally, we determined the rheumatoid factor (RF), C-reactive protein (CRP), antinuclear antibodies (ANAs), anti-cyclic citrullinated peptide (anti-CCP), and HLA-27, as well as performed magnetic resonance imaging, bilateral neuro conduction, and electromyography tests in selected patients per request of the rheumatologist.

### 2.5. Outcomes

#### 2.5.1. Post-CHIK Chronic Rheumatism (pCHIK-CR)

Post-CHIK chronic rheumatism (pCHIK-CR) was defined as an abnormal GALS examination with persistence of joint and extra-articular symptoms for more than three months after the onset of CHIKV disease or the development of a specific immune-mediated inflammatory pathology during follow-up [41,42]. We categorized patients with pCHIK-CR based on the following definitions: rheumatoid arthritis (according to ECR/EULAR 2010 criteria) [60], axial or peripheral spondylarthritis (based on ASAS 2011 criteria) [61], systemic lupus erythematosus (following ACR/EULAR 2019 criteria) [62], post-viral arthritis (confirmed by joint pain and fluctuation of joint margins in one or more joints) [63], post-viral arthralgia (documented by joint pain at the margin level without swelling) [63], and soft tissue rheumatism (identified by the presence of tenosynovitis, bursitis, fasciitis, noninflammatory localized pain, enthesitis, or fibromyalgia based on ACR 2011 criteria) [64]. We classified participants with an abnormal GALS examination but not meeting the pCHIK-CR criteria as non-inflammatory pain likely degenerative (NIP-LD) cases. Participants with a normal GALS examination who did not meet the pCHIK-CR criteria were classified as cases without rheumatic manifestations (Wo-RM).

#### 2.5.2. Quality of Life (QoL)

We evaluated the QoL using the SF-36v2™ questionnaire [65]. This questionnaire includes 36 items assessing eight domains of health status: physical functioning, physical role, bodily pain, general health, vitality, social functioning, emotional role, and mental health. These domains are classified into two main components: mental and physical [66,67,68]. The SF-36 score ranges from 0 to 100, with higher scores indicating better quality of life. We categorized the scores for each domain and component into three levels based on standardized data from the general US population [69]: ≤40 as “Well Below”; between 40 and 44 as “Below”; and ≥45 as “Same or Better” than the mean population score [42]. We used the PROCoRE Smart Measurement^®^ System software, version 2.1.2.20088 for data analysis and defined impaired QoL if any component (mental or physical) was classified into the “Well Below” category.

#### 2.5.3. Chronic Fatigue (CF)

We used the Fatigue Severity Scale (FSS) to evaluate fatigue [59,70]. This scale uses a Likert-type format with seven levels to assess physical, social, and motivational dimensions of fatigue, where higher scores indicate greater severity. We defined fatigue as a total FSS score greater or equal to 36 points [71] and chronic fatigue (CF) in participants reporting fatigue lasting for more than 6 months [48].

### 2.6. Data Analysis

We described continuous variables by estimating the mean and standard deviation (SD) or the median and interquartile range [IQR] for those not normally distributed, according to the Shapiro-Wilk test. We calculated their absolute and relative frequencies (percentages) for discrete variables. We contrasted means and medians between groups using a student’s *t*-test or analysis of variance (ANOVA) and the sum of ranks test, respectively, and differences in proportions by using the chi-square test and, alternatively, the Fisher’s exact test whenever the expected counts in contingency tables were less than five. We adjusted for multiple comparisons using the Bonferroni correction. The GALS examination’s positive and negative predictive values and 95% confidence intervals (95%CI) for pCHIK-CR, as determined by the rheumatologist, were estimated using the exact binomial method. We estimated the correlation between the HAQ-DI and MSQ scales using Spearman’s correlation coefficient (rs) and their agreement (dichotomized around their medians) with patients’ classification (the extreme classes: pCHIK-CR versus Wo-RM) using Cohen’s Kappa. Finally, we evaluated the association between QoL and chronic fatigue with joint involvement (pCHIK-CR and NIP-LD versus Wo-RM) by using multinomial logistic regression, adjusting for age, sex, and comorbidities. The analyses were conducted using Stata version 12.0 (Stata Corp., College Station, TX, USA).

## 3. Results

**Figure 1:**
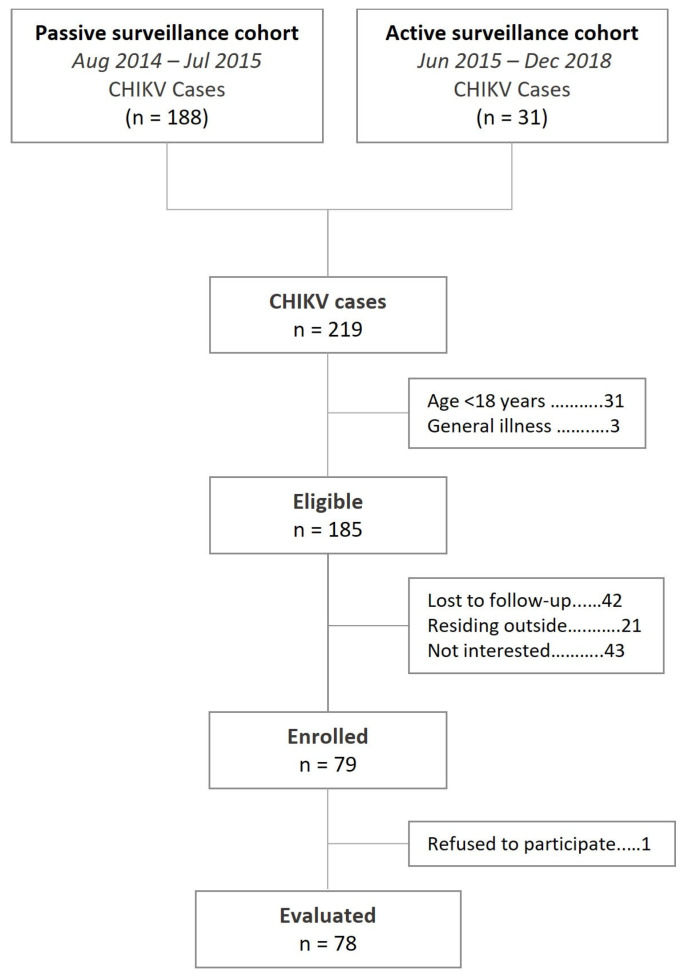
Flowchart of the re-contact and enrolment processes.

We identified 219 cases of CHIKV infection from both data sources that occurred between November 2014 and December 2015, of which 185 (85.5%) were eligible for re-contacting (Figure 1). Upon completing the protocol of re-contact, 42 (22.7%) patients could not be reached, 21 (11.4%) reported residing outside the Metropolitan Area of Bucaramanga, and 43 (23.2%) declined the invitation to participate. Only one of the enrolled participants declined to attend the follow-up evaluation; therefore, the study’s final sample consisted of 78 individuals. Enrolled participants were older than those who could not be contacted or did not accept to participate (35.0 versus 24.0, *p* = 0.001), but the sex distribution was comparable (*p* = 0.114). The median age of the sample at CHIKV symptoms’ onset was 30.0 years (IQR = 21.0 years) and included 47 women (60.3%). The mean duration of the symptoms during the acute phase of the infection was 2.8 days (SD = 1.5 days): 92.3% and 97.5% of the cases reported arthralgia and fatigue, respectively. None of the participants reported a medical history of articular disease before the CHIKV infection; however, 1.3% and 3.9% had prevalent diagnoses of diabetes and cardiovascular disease, respectively (Table 1).

The median time between the disease’s onset and the follow-up evaluation was 7.7 years, at which 14.1% (95%CI: 7.3–23.8) of participants were classified as cases of pCHIK-CR, 41.0% (95%CI: 30.0–52.7) as cases of NIP-LD, and 44.9% (95%CI: 33.6–56.6) did not have any clinical manifestation of rheumatic disease (Wo-RM; Table 2). Among pCHIK-CR patients, the three most frequent clinical patterns that the rheumatologist identified were post-viral oligo/polyarthritis (36.4%), post-viral arthralgia (27.2%), and fibromyalgia (18.2%). Most pCHIK-CR cases reported arthralgia (72.7%), with a median pain scale score of 7.0 (IQR = 2.5). The knees (62.5%), hands (50.0%), and feet (37.5%) were the most affected joints. Additionally, 27.3% of pCHIK-CR cases reported stiffness, with a median MSQ score of 8.0 (IQR = 5.0). The GALS screening evaluation’s estimated positive and negative predictive values were 25.6% (95% CI: 13.5–41.2) and 100.0% (95% CI: 47.8–100.0), respectively, compared to the rheumatologist’s criterion.

Patients with pCHIK-CR and NIP-LD were younger and predominantly female compared to Wo-RM cases (Table 3). Joint stiffness and swelling were more often reported by cases of pCHIK-CR than NIP-LD and Wo-RM; however, cases of NIP-LD showed the highest frequency of gait limitation. There was no difference between groups in terms of comorbidities or obesity. Still, patients with pCHIK-CR reported higher dexamethasone use (at any time post-CHIKV infection) than cases of NIP-LD and Wo-RM: 18.2% versus 3.1% and 0.0%, respectively. Although the grip strength was similar in patients with pCHIK-CR and NIP-LD, it was significantly lower compared to Wo-RM cases: 19.1 kg, 18.1 kg, and 32.1 kg, respectively. The median MSQ and HAQ-DI scores showed statistically significant gradients across groups (with the lowest values observed among Wo-RM cases) and a positive correlation (rs = 0.69, *p* < 0.001). Once dichotomized around their medians, the HAQ-DI scale showed a stronger concordance with the study outcome (considering the extreme classes: pCHIK-CR versus Wo-RM) than the MSQ scale (kappa = 0.54, *p* < 0.001; and kappa = 0.32, *p* = 0.013, respectively).

**Figure 2:**
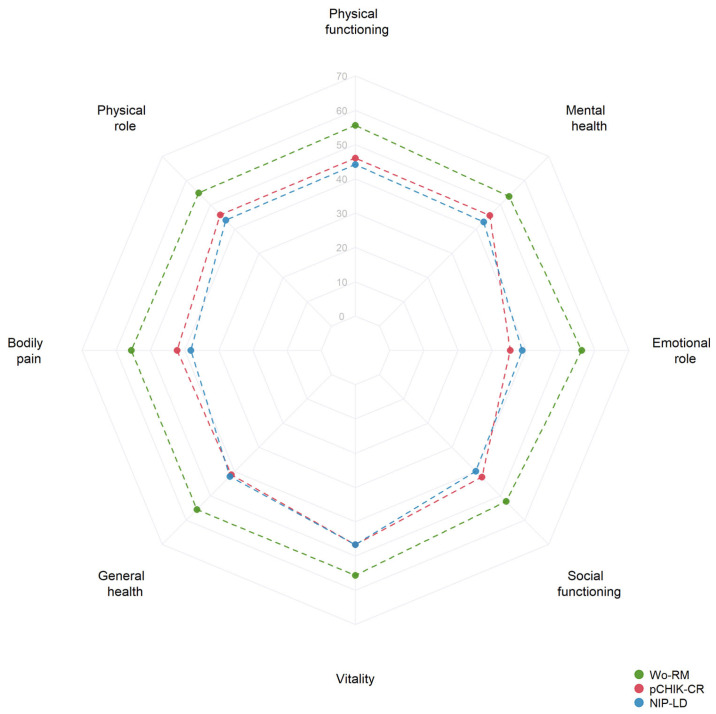
Quality of life was assessed using the SF-36 questionnaire during the follow-up evaluation by a clinical pattern of post-CHIK chronic rheumatism. The medians of the eight domains of the SF-36 questionnaire are represented in a radar graph by the clinical patterns of post-CHIK chronic rheumatism. Wo-RM: Without rheumatic manifestations; pCHIK-CR: Post-CHIK Chronic Rheumatism; NIP-LD: Non-inflammatory pain likely degenerative.

Figure 2 illustrates the quality of life (QoL) assessed with the SF-36 questionnaire across groups of participants according to the pattern of chronic rheumatism. Patients with pCHIK-CR and NIP-LD showed significantly lower median scores across the eight domains of the SF-36 compared to Wo-RM cases (Table 4). Participants with affected QoL were significantly more likely to have pCHIK-CR than Wo-RM (adjusted OR = 11.4, 95%CI: 1.8–73.7). Similarly, affected QoL was associated with increased odds of being a NIP-LD case than Wo-RM (adjusted OR = 6.8, 95% CI: 1.8–25.3). The prevalence of chronic fatigue gradually increased from 8.6% in patients Wo-RM to 25.0% and 54.6% among NIP-LD and pCHIK-CR cases, respectively. Chronic fatigue was significantly associated with pCHIK-CR (adjusted OR = 19.0, 95% CI: 2.46–146.42) but not with NIP-LD (adjusted OR = 2.92, 95% CI: 0.62–13.65) considering Wo-RM as the reference group.

## 4. Discussion

Based on the harmonization and extension of follow-up of two cohorts of patients diagnosed with CHIKV infection between 2014 and 2015, we estimated a 14.1% incidence of post-chikungunya chronic rheumatism (pCHIK-CR) after 7.7 years from symptoms onset. The rheumatological evaluation showed a predominance of the post-viral oligo/polyarthritis pattern among pCHIK-CR cases. Additionally, 41.0% and 44.9% of all participants were classified as non-inflammatory pain likely degenerative (NIP-LD) cases and without rheumatic manifestations (Wo-RM), respectively, and no patient fulfilled criteria for rheumatoid arthritis, lupus, or spondyloarthropathy. The Health Assessment Questionnaire-Disability Index (HAQ-DI) and the Musculoskeletal Stiffness Questionnaire (MSQ) were positively correlated; however, the first showed a higher concordance with the pCHIK-CR status than the latest. Patients with impaired quality of life (QoL) or chronic fatigue were more likely classified as cases of pCHIK-CR than Wo-RM. Finally, the Gait, Arms, Legs, and Spine (GALS) screening used to identify pCHIK-CR cases by general physicians demonstrated positive and negative predictive values of 25.6% and 100.0%, respectively.

Multiple cohorts have studied the degree of long-term joint involvement in patients with CHIKV infection [46,72,73,74,75,76]. However, the understanding of the pathogenesis of chronic rheumatic symptoms remains limited. However, in vitro studies have shown that CHIKV exhibits tropism for endothelial and epithelial cells, fibroblast, and monocyte-derived macrophages, exerting a strong cytopathic effect in human-infected cells, similar to other alphaviruses [18]. It has been demonstrated that CHIKV has a specific tropism for muscle, joint, and skin fibroblasts, which may explain the joint pain experienced by patients during the acute phase of the infection [77]. Furthermore, the high and persistent viremias reported during the acute phase may be an effect of the capability of CHIKV to modulate the induction of type I interferons (IFNs) and the effector molecules of their signaling pathway, specifically, the non-structural protein 2 (nsP2) of CHIKV and other alphaviruses is a potential immune modulator that inhibits the infected cell antiviral state [2,18,37,78]. A supporting finding of this was the report of the presence of RNA and viral proteins in synovial macrophages obtained from a patient after 18 months of CHIKV infection [21]. Another hypothesis of the long-lasting rheumatic symptoms is the maintained production of pro-inflammatory molecules by the synovial tissue cells. The levels of the chemokines MCP-1, MIG, IP-10, and IL-8 have been reported elevated during CHIKV infection. These molecules are involved in recruiting macrophages, monocytes, NK, and T Cells to the affected joints [18,31,38]. Moreover, the presence of MCP-1, IL-6, and IL-8 may facilitate the differentiation of monocytes into osteoclasts, which enhances bone resorption and exacerbates joint damage during CHIKV infection [18,39]. The understanding of the mechanism involved in the imbalance between antiviral response and inflammation resolution is crucial for the development of therapies for reducing acute symptoms and preventing the development of rheumatic manifestations.

Our analysis, based on harmonized data from two of the lengthiest follow-up studies conducted in Latin America, revealed that about one out of seven patients experience pCHIK-CR up to 7.7 years after the acute phase of CHIKV infection; however, comparing this estimate to those from other cohorts is challenging due to methodological heterogeneity across studies and the tendency for these symptoms to fade over time. The latest could be attributable, among other reasons, to the natural progression of the disease, the susceptibility of the exposed population, or the use of treatments aimed at modulating the inflammatory response. For example, in the Reunion Island cohort, which also had a long-term follow-up, the prevalence of chronic inflammatory rheumatism decreased from 45.9% to 10.7% at 3 and 13 years post-CHIKV infection [15,72].

Previous studies conducted in Colombia documented a high prevalence of joint pain or pCHIK-CR within the first and second years after CHIKV infection, with rates of 58.5% and 25.4%, respectively [74,75]. However, these studies relied on retrospective data or self-reporting through telephone surveys. Similarly, a cohort study conducted in Capitanejo, Colombia, found a 25.0% (95%CI: 14.4–38.4) incidence of pCHIK-CR, diagnosed by a rheumatologist, two years post-infection [14]. Despite using the same definition of pCHIK-CR, the incidences could not be directly compared because of the length of follow-up (two years versus seven years).

In addition, a survey conducted in 2017 across five Colombian cities estimated CHIKV seroprevalence rates ranged from 9% to 73% during the outbreak [6,7]. Extrapolating from our results, which indicate a 14.1% incidence of chronic rheumatism seven years after acute infection, we estimate that between 412,872 and 3,356,959 Colombians could be affected by this condition, using the 2015 estimate of 32,614,000 Colombian adults as reference [79].

The clinical characterization of pCHIK-CR cases by trained rheumatologists could help more reliably identify chronic chikungunya disease. Our study showed that the most common clinical patterns of pCHIK-CR were post-viral oligo/polyarthritis, post-viral arthralgia, and fibromyalgia. In the Reunion Island cohort, patients with pCHIK-CR exhibited more severe clinical patterns, such as rheumatoid arthritis, spondylarthritis, and psoriatic arthritis in 64.7%, 23.5%, and 11.8% of the cases, respectively [72]. These differences in patterns and severity across studies could be partially attributed to differences in the CHIKV genotypes that circulated during the outbreaks in Reunion Island and the Americas [80,81]. Studies in animal models have shown that the Asian genotype, which circulated in the Americas, has weaker replication or competitive fitness and induces moderate joint edema and swelling, with a weaker proinflammatory Th1 response and natural killer cell activity compared to the ECSA genotype [82,83]. These differences should be taken into account, as they might contribute to variations in the clinical evolution observed during the chronic phase in humans. Furthermore, the prevalence of symptoms, chronic inflammatory rheumatism, and other pathologic outcomes may differ in Western, African, or Asian cohorts.

Another factor that could influence the incidence of rheumatologic findings and clinical patterns is baseline susceptibility to inflammatory/autoimmune disorders in different populations. According to epidemiological studies, the prevalence of rheumatologic diseases by the time of the introduction of Chikungunya in Colombia was lower compared to other populations like Europeans, Asians, and even other native American people [84,85]. We should highlight that in our cohorts, none of the cases that met the criteria for pCHIK-CR have undergone treatment with disease-modifying anti-rheumatic drugs (DMARDs). This observation could be due to several reasons. One possibility is that the inflammation shown by these patients after the acute phase is less severe compared to other cohorts with the same diagnosis. On the other hand, we cannot dismiss the possibility that the low use of DMARDs is due to limited access to healthcare services in this population or the lack of evidence-based guidelines for using these types of therapies in this disease.

The involvement of joints, as indicated by higher HAQ-DI scores, showed agreement with pCHIK-CIR when contrasted against Wo-RM cases. This is consistent with previous findings from a cohort of CHIKV-infected patients who did not use DMARDs during 24-m [20,22,23,27,29,31,32,33,86,87,88]. Additionally, Watson et al. [89] reported elevated HAQ-DI scores in patients experiencing chronic arthralgia, with a strong correlation between this index and the degree of joint pain and tenderness after three years of follow-up. These findings suggest significant impairment among patients with pCHIK-CR, potentially affecting their ability to perform daily life activities such as dressing, arising, walking, reaching, self-hygiene, and handling objects.

Patients with chronic articular symptoms not only experienced impaired quality of life (QoL) as measured by the SF-36v2 questionnaire but also chronic fatigue. Compared to Wo-RM cases, patients with pCHIK-CR and NIP-LD had lower SF-36 physical and mental component scores; however, there were no significant differences in QoL between pCHIK-CR and NIP-LD cases. An impaired QoL was associated with the outcomes of pCHIK-CR and NIP-LD when Wo-RM cases were taken as a reference. This finding suggests that CHIKV-induced rheumatologic involvement could lead to a disease burden like other non-inflammatory rheumatologic diseases. Previous studies have also shown that patients exposed to CHIKV reported lower SF-36 scores compared with those not exposed, with the impairment of QoL persisting even at 30 and 72 months of follow-up [47,90]. Furthermore, it was reported that CHIKV cases who recovered from rheumatologic symptoms showed higher SF-36 scores compared to those with persistent joint involvement two years post-infection [45,46].

In our harmonized cohorts of CHIKV-exposed patients, we found that 21.8% of them experienced chronic fatigue during the follow-up examination, with a higher frequency among pCHIK-CR cases. After adjusting for age, sex, and comorbidities, we observed a significant association between chronic fatigue and pCHIK-CR. The literature supports the relationship between acute infections and chronic fatigue in cases such as dengue, Ross River virus, Epstein-Barr virus, and cytomegalovirus, among others [71,91,92]. In addition to the rheumatologic involvement in chronic CHIKV disease, chronic fatigue could reflect other persistent symptoms accompanying this condition [21,22]. Manimunda et al. [49] reported fatigue in 34% of CHIKV infection cases at one month and in 13% at ten months of follow-up, while in the TELECHIK cohort, 34.3% of patients presented idiopathic chronic fatigue [48].

Impaired quality of life and chronic fatigue may be an effect of the pCHIK-CR symptoms and could be causes of the immune dysregulation induced by the CHIKV pro-inflammatory response. Moreover, the cytokines IL-6, TNF-α, and IL-8 have been suggested as molecules involved in depression, specifically in chronic fatigue and sleep disorders [93].

The rheumatologic characterization of patients with chronic CHIKV infection involved two stages. First, a physician trained in musculoskeletal semiology performed an evaluation using the GALS assessment. Then, a trained rheumatologist conducted a second assessment, mimicking typical healthcare pathways. This approach aimed to provide valuable information to identify patients needing specialized assessment rather than just primary care. Our findings suggest that a primary healthcare approach based on the GALS assessment could effectively rule out most patients without joint involvement while correctly identifying just one out of four pCHIK-CR cases. Currently, comprehensive healthcare pathways for infectious diseases such as chikungunya succinctly address the management of chronic cases [94]. Therefore, our results could shape recommendations for disease management and improve timely access to therapeutic interventions among patients with CHIKV infection. This is particularly relevant in countries such as Colombia, where exclusively the Asian genotype [81,95] was circulated, and a wide range of attack rates were observed during the 2014–2015 outbreak [6,7]. Furthermore, the recent increase in CHIKV cases in the Americas, particularly in Brazil and Paraguay, has been associated with a higher number of deaths attributable to the virus, mainly due to the ECSA genotype [4,96,97].

Our study has some strengths worth mentioning. Firstly, the extended follow-up period allowed us to observe the long-term progression of post-chikungunya chronic rheumatism (pCHIK-CR). Secondly, the involvement of trained rheumatologists in clinical evaluations enhances the accuracy of diagnosing clinical patterns of chronic rheumatism. Thirdly, we assessed the performance of the GALS screening tool, highlighting its potential utility for initial assessments in primary care during the chronic phase of the disease. Our study also has limitations. Firstly, the cases of chikungunya infection were identified in a single municipality in Colombia, which could limit the generalizability of the findings to other populations and epidemiological contexts. Secondly, the fact that enrolled patients were older than those eligible might have overestimated the incidence of pCHIK-CR. Thirdly, the total sample size reached, even after harmonizing data from two cohorts, might have limited the power to detect associations and the accuracy of our estimates. Finally, we did not assess the quality of life and chronic fatigue during the acute phase of CHIKV infection, so our findings can only be considered correlational.

## 5. Conclusions

Our results showed that about one out of seven patients with CHIKV infection developed symptoms and signs compatible with chronic rheumatism (pCHIK-CR) almost eight years post-disease onset. This condition leads to a significant impairment of QoL and a high prevalence of chronic fatigue, comparable to that observed in non-inflammatory rheumatic diseases. The comprehensive approach we implemented for characterizing CHIKV cases could be a valuable tool for assessing the disease burden, facilitating the design of clinical trials, and developing patient management strategies; however, these findings require further replication in the context of larger cohorts.

Given the gaps in the understanding of CHIKV pathogenesis, further clinical characterization studies should be accompanied by the assessment of immunological markers that can predict the disease progression. Specifically, research involving synovial membrane biopsies is needed to evaluate predisposing factors associated with long-lasting rheumatic complications.

## Figures and Tables

**Table 1:** Characteristics of patients during the acute phase of CHIKV infection (2014–2015), categorized by the source of information.

Characteristic, *n* (%)	Passive Surveillance Cohort (*n* = 65)	Active Surveillance Cohort (*n* = 13)	All (*n* = 78)
Sex			
Male	23 (35.4)	8 (61.5)	31 (39.7)
Female	42 (64.6)	5 (38.5)	47 (60.3)
Age (years)			
<18	9 (13.8)	8 (61.5)	16 (20.5)
18–30	21 (32.3)	0 (0.0)	22 (28.2)
31–45	25 (38.5)	5 (38.5)	30 (38.5)
≥46	10 (15.4)	0 (0.0)	10 (12.8)
Days of illness, Mean (SD)	2.8 (1.6)	3.0 (1.2)	2.8 (1.5)
Symptoms			
Arthralgia	62 (95.4)	10 (76.9)	72 (92.3)
Myalgia	54 (83.1)	11 (84.6)	65 (83.3)
Fatigue	65 (100.0)	11 (84.6)	76 (97.4)
Skin rash	53 (81.5)	10 (76.9)	63 (80.8)
Headache	62 (95.4)	11 (84.6)	73 (93.6)
Nauseas	36 (55.4)	3 (23.1)	39 (50.0)
Medical history			
Diabetes mellitus	1 (1.5)	0 (0.0)	1 (1.3)
Renal disease	0 (0.0)	0 (0.0)	0 (0.0)
Liver disease	0 (0.0)	0 (0.0)	0 (0.0)
Cardiovascular disease	3 (4.6)	0 (0.0)	3 (3.9)
Articular disease	0 (0.0)	0 (0.0)	0 (0.0)

**Table 2:** Clinical patterns of chronic rheumatism during the follow-up evaluation.

Clinical Patterns	*n* (%)
Post-CHIK Chronic Rheumatism	11 (14.1)
Rheumatoid arthritis	0 (0.0)
Post-viral oligo/polyarthritis	4 (36.4)
Post-viral arthralgia	3 (27.2)
Soft tissue rheumatism	
Tenosynovitis	0 (0.0)
Fibromyalgia	2 (18.2)
Fasciitis	1 (9.1)
Enthesitis	1 (9.1)
Non-inflammatory pain likely degenerative	32 (41.0)
Without rheumatic manifestations	35 (44.9)

**Table 3:** Characteristics of participants during the follow-up evaluation, categorized by clinical pattern of chronic rheumatism.

Characteristic	pCHIK-CR (*n* = 11)	NIP-LD (*n* = 32)	Wo-RM (*n* = 35)	*p*
Female, *n* (%)	9 (81.2)	24 (75.0) ^b^	14 (40.0)	0.004
Age (years)	45.0 [15.0] ^a^	40.5 [16.5] ^b^	30.0 [20.0]	0.004
Symptoms, *n* (%)				
Myalgia	5 (45.5)	20 (62.5)	13 (37.1)	0.113
Paresthesia	8 (72.7)	21 (65.6)	14 (40.0)	0.059
Stiffness	2 (18.2)	1 (3.1)	0 (0.0)	0.025
Limitation of gait	4 (36.4) ^a^	18 (56.3) ^b^	2 (5.7)	0.000
Joint swelling	7 (63.4) ^a^	13 (40.6) ^b^	1 (2.9)	0.000
Medical history, *n* (%)				
Arthritis	1 (9.1)	1 (3.1)	0 (0.0)	0.136
Osteoarthrosis	1 (9.1)	1 (3.1)	0 (0.0)	0.136
Hypertension	3 (27.3)	5 (15.6)	3 (8.6)	0.239
Diabetes	1 (9.1)	1 (3.1)	1 (2.9)	0.522
Chronic renal disease	1 (9.1)	0 (0.0)	0 (0.0)	0.141
Liver disease	0 (0.0)	1 (3.1)	0 (0.0)	0.551
Medication use, *n* (%)				
Dexamethasone	2 (18.2)	1 (3.1)	0 (0.0)	0.025
Prednisolone	0 (0.0)	2 (6.3)	0 (0.0)	0.429
Methotrexate	0 (0.0)	1 (3.1)	0 (0.0)	0.551
Body mass index (kg/m^2^)	31.0 [9.8]	26.7 [8.7]	27.0 [6.5]	0.584
Handgrip (kg)	19.1 [16.5] ^a^	18.1 [14.2] ^b^	32.1 [22.0]	0.043
HAQ-DI score	0.6 [1.5] ^a^	0.5 [0.6] ^b^	0.0 [0.1]	0.000
HAQ-DI categories				
Dressing and grooming	0.0 [1.0] ^a^	0.0 [0.0]	0.0 [0.0]	0.039
Arising	1.0 [1.0] ^a^	0.5 [1.0] ^b^	0.0 [0.0]	0.000
Eating	0.0 [0.0]	0.0 [0.5]	0.0 [0.0]	0.195
Walking	0.0 [2.0] ^a^	1.0 [1.0] ^b^	0.0 [0.0]	0.000
Hygiene	0.0 [1.0] ^a^	0.0 [0.0]	0.0 [0.0]	0.008
Reaching	1.0 [1.0] ^a^	0.0 [1.0] ^b^	0.0 [0.0]	0.001
Grip	0.0 [1.0] ^a^	0.0 [1.0] ^b^	0.0 [0.0]	0.012
Usual activities	0.0 [2.0] ^a^	0.0 [1.0] ^b^	0.0 [0.0]	0.002
MSQ score (%)	22.8 [40.3] ^a^	30.1 [26.9] ^b^	0.0 [5.0]	0.000

The values in square brackets denote the interquartile range [IQR]. pCHIK-CR: Post-CHIK Chronic Rheumatism; NIP-LD: Non-inflammatory pain likely degenerative; Wo-RM: Without rheumatic manifestations. ^a^ *p* < 0.050 for the comparison between pCHIK-CR and Wo-RM. ^b^ *p* < 0.050 for the comparison between NIP-LD and Wo-RM.

**Table 4:** Quality of life and fatigue were assessed during the follow-up evaluation using a clinical pattern of chronic rheumatism.

Assessment	pCHIK-CR (*n* = 11)	NIP-LD (*n* = 32)	Wo-RM (*n* = 35)	*p*
SF-36				
Physical component	46.1 [16.1] ^a^	43.5 [17.2] ^b^	56.7 [7.8]	0.001
Physical functioning	46.1 [19.1] ^a^	44.2 [19.1] ^b^	55.6 [3.8]	0.000
Physical role	45.9 [27.0]	43.7 [18.0] ^b^	54.9 [13.5]	0.004
Bodily pain	42.2 [21.0] ^a^	38.2 [17.0] ^b^	55.6 [6.5]	0.000
General health	41.3 [19.0] ^a^	42.0 [20.7] ^b^	55.6 [11.9]	0.003
Mental component	39.8 [16.0] ^a^	43.2 [13.8] ^b^	53.0 [13.0]	0.043
Vitality	46.7 [8.9] ^a^	46.7 [8.9] ^b^	55.6 [11.9]	0.000
Social functioning	42.3 [15.0] ^a^	39.8 [15.0] ^b^	52.3 [10.0]	0.006
Emotional role	35.3 [24.4] ^a^	38.8 [20.9] ^b^	56.2 [13.9]	0.006
Mental health	45.6 [18.3] ^a^	43.0 [15.7]	53.5 [15.7]	0.014
Impaired QoL, *n* (%)	6 (54.6) ^a^	19 (59.4) ^b^	5 (14.3)	0.000
FSS score	44.0 [21.0] ^a^	35.5 [33.0] ^b^	15.0 [22.0]	0.002
Chronic Fatigue, *n* (%)	6 (54.6) ^a^	8 (25.0)	3 (8.6)	0.005

The values in square brackets denote the interquartile range [IQR]. pCHIK-CR: Post-CHIK Chronic Rheumatism; NIP-LD: Non-inflammatory pain likely degenerative; Wo-RM: Without rheumatic manifestations. Chronic fatigue was defined as an FSS score ≥ 36 lasting over six months. Quality of life (QoL) impairment was defined as a score ≤ 40 in any component of the SF-36 questionnaire. ^a^ *p* < 0.050 for the comparison between pCHIK-CR and Wo-RM. ^b^ *p* < 0.050 for the comparison between NIP-LD and Wo-RM.

## Data Availability

The data supporting the findings of this study are available upon reasonable request. Due to privacy and ethical restrictions, the data cannot be made publicly available.
